# Integrin β1 orchestrates the abnormal cell-matrix attachment and invasive behaviour of E-cadherin dysfunctional cells

**DOI:** 10.1007/s10120-021-01239-9

**Published:** 2021-09-05

**Authors:** Joana Figueiredo, Rui M. Ferreira, Han Xu, Margarida Gonçalves, André Barros-Carvalho, Janine Cravo, André F. Maia, Patrícia Carneiro, Céu Figueiredo, Michael L. Smith, Dimitrije Stamenović, Eurico Morais-de-Sá, Raquel Seruca

**Affiliations:** 1 i3S-Instituto de Investigação e Inovação em Saúde, University of Porto, Rua Alfredo Allen 208, 4200-135 Porto, Portugal; 2 Institute of Molecular Pathology and Immunology of the University of Porto (IPATIMUP), 4200-135 Porto, Portugal; 3 Department of Biomedical Engineering, Boston University, Boston, MA 02215 USA; 4 Institute for Molecular and Cell Biology (IBMC), University of Porto, 4200-135 Porto, Portugal; 5 Instituto de Ciências Biomédicas de Abel Salazar (ICBAS), Universidade do Porto, 4050-313 Porto, Portugal; 6 Medical Faculty of the University of Porto, 4200-319 Porto, Portugal; 7 Division of Material Science and Engineering, Boston University, Brookline, MA 02215 USA

**Keywords:** E-cadherin, Hereditary diffuse gastric cancer, Extracellular matrix, Invasion, Integrin β1

## Abstract

**Background:**

Tumour progression relies on the ability of cancer cells to penetrate and invade neighbouring tissues. E-cadherin loss is associated with increased cell invasion in gastric carcinoma, and germline mutations of the E-cadherin gene are causative of hereditary diffuse gastric cancer. Although E-cadherin dysfunction impacts cell–cell adhesion, cell dissemination also requires an imbalance of adhesion to the extracellular matrix (ECM).

**Methods:**

To identify ECM components and receptors relevant for adhesion of E-cadherin dysfunctional cells, we implemented a novel ECM microarray platform coupled with molecular interaction networks. The functional role of putative candidates was determined by combining micropattern traction microscopy, protein modulation and in vivo approaches, as well as transcriptomic data of 262 gastric carcinoma samples, retrieved from the cancer genome atlas (TCGA).

**Results:**

Here, we show that E-cadherin mutations induce an abnormal interplay of cells with specific components of the ECM, which encompasses increased traction forces and Integrin β1 activation. Integrin β1 synergizes with E-cadherin dysfunction, promoting cell scattering and invasion. The significance of the E-cadherin-Integrin β1 crosstalk was validated in *Drosophila* models and found to be consistent with evidence from human gastric carcinomas, where increased tumour grade and poor survival are associated with low E-cadherin and high Integrin β1 levels.

**Conclusions:**

Integrin β1 is a key mediator of invasion in carcinomas with E-cadherin impairment and should be regarded as a biomarker of poor prognosis in gastric cancer.

**Supplementary Information:**

The online version contains supplementary material available at 10.1007/s10120-021-01239-9.

## Introduction

E-cadherin is an essential molecule for epithelial homeostasis by regulating epithelial architecture and tissue integrity [1]. In cancer, genetic and epigenetic alterations in the E-cadherin gene (*CDH1*) or aberrant protein expression are frequent and result in loss of cell–cell adhesion, increased cell invasion and metastasis [2]. In the hereditary form of diffuse gastric cancer, known as hereditary diffuse gastric cancer syndrome (HDGC), inactivating germline alterations of the *CDH1* gene are causative events [3, 4]. HDGC is a highly penetrant cancer syndrome characterized by multiple *foci* of isolated malignant cells that invade widely through the gastric wall [4, 5]. Precursor lesions of invasive gastric cancer have been identified in *CDH1* mutation carriers as in situ signet ring cell carcinoma (SRCC) or pagetoid spread of signet ring cells below the preserved epithelium of glands [6, 7]. These early gastric lesions are a hallmark of the disease and provide unique evidence that, in the initial steps of the neoplastic process, E-cadherin dysfunctional cells lack cellular cohesion but maintain a close contact with the basement membrane—a specialized extracellular matrix (ECM) that supports and fine-tunes cellular functions [6, 7].

Despite progress in the characterization of invasive gastric cancer cells, the mechanisms underlying their aggressive spreading remain largely unknown. We hypothesize that E-cadherin mutations associated with HDGC lead to aberrant integrin activation and signalling, initiating a specific mechano-transduction pathway at the early steps of invasion. In the last few years, data have emerged demonstrating a complex interplay between cell–cell junctions and cell-ECM adhesion [8, 9]. While cadherins are central for cell–cell contacts, integrins are the main regulators of the cell-ECM crosstalk, working as mechanical transducers and as signalling molecules [1, 10, 11]. In homeostasis, the relationship between cadherins and integrins is tightly controlled, whereas in cancer this crosstalk is dysregulated and highly associated with invasion and metastasis [11–13].

In this study, we dissected the cascade of events occurring at the interface of E-cadherin dysfunctional cells with the ECM. Our strategy encompassed a high-content screening approach coupled with molecular interaction networks to identify ECM components and receptors relevant for adhesion of E-cadherin mutant cells. Functional significance of the findings was subsequently addressed through a set of in vitro and in vivo assays, as well as transcriptomic data of gastric carcinoma samples, retrieved from the cancer genome atlas (TCGA). Overall, our results demonstrate that E-cadherin loss modifies physical and biochemical features of the cell-matrix interaction, and pinpoint Integrin β1 as the key player in cadherin-mediated invasion. This data highlights ECM-specific receptors, such as integrins, as novel biomarkers of poor prognosis in gastric cancer with E-cadherin impairment.

## Materials and methods

### Plasmids

E-cadherin variants A634V (c.1901C > T), R749W (c.2245C > T) and V832M (c.2494G > A) were induced by site-directed mutagenesis in the entry vector CDH1pENTR 221 (Clone ID: IOH46767, Invitrogen, Grand Island, NY, USA) and subcloned into the pEF6/Myc-His vector (Invitrogen) by LR recombination [14]. The corresponding empty vector (Mock) was constructed by restriction of the CDH1pEF6/Myc-His with BsrGI (Biolabs, Ipswich, MA, USA).

### Cell culture and transfection

AGS cell line (gastric adenocarcinoma, ATCC number CRL-1739) was maintained and transfected as described in Supplementary Materials and Methods.

### ECM microarrays

The cell-matrix adhesion profile of AGS cells transfected with the wild-type and the A634V, R749W and V832M E-cadherin variants was determined using a MicroMatrix™ 36 cell culture system (MicroStem). A suspension of 2.5 × 10^5^ cells were seeded on the array slides. Slides were incubated at 37 °C under 5% CO_2_ humidified air for 48 h to allow cell attachment. Cells were washed in PBS and fixed in ice-cold methanol for 20 min. Nuclear staining was achieved with a 1 µg/ml DAPI solution. Image acquisition was performed on an IN Cell Analyzer 2000 (GE Healthcare) with a Nikon 10x/0.45NA objective. Nuclei segmentation was achieved with ilastik [15], and CellProfiler [16] was subsequently used for image analysis and quantification. Similarity between cell-matrix attachment profiles was determined through Pearson’s correlation distances. Significant differences in the number of wild-type and mutant adherent cells were evaluated using linear discriminant analysis effect size (LEfSe). ECM compositions with LDA > 4 and *P* < 0.05 were considered significantly enriched.

### ECM-integrin network analysis

A consensus list of receptors and corresponding ECM ligands was retrieved from published records [17, 18]. Based upon this data, all possible physical interactions between integrins and ECM compositions assayed in the array were analysed. The network diagram was created using Cytoscape (version 3.1.0) [19]. Relative frequency of each specific integrin subunit (β and α) was calculated considering the total number of interactions with ECM compositions that induced significant alterations in mutant attachment (estimated using LEfSe analysis).

### Micropattern traction microscopy

Micropattern traction microscopy was performed as previously described [20]. A detailed description is provided at Supplementary Materials and Methods.

### Western blotting

Cell lysates were analysed as described by Figueiredo et al. [14].

### Matrigel invasion assays

Cell invasive abilities were assessed using matrigel invasion inserts suitable for 24-well-plates (Corning BioCoat). Detailed protocol available at Supplementary Materials and Methods.

### Cell topology analysis

Cellular distribution patterns were examined using fluorescence images of DAPI-stained cells. Image denoising and nuclei segmentation were performed in each image by applying the Otsu method and the Moore-Neighbor tracing algorithm, modified by Jacob’s stopping criteria [21]. Nuclei geometric centres were computed and connected using the Delaunay triangulation algorithm. Triangles composing cellular networks were analysed for features such as area and edges length.

### *Drosophila* strains and genetic manipulations

A transgenic *Drosophila* model based on the Gal4/UAS system was used to target gene expression in fly tissues. For specific expression in border cells, we generated flies containing UAS-hE-cad WT or UAS-hE-cad R749W together with UAS-mCherry, which were all expressed using GAL4 driven from a border cell-specific promoter (*slbo-Gal4,* BDSC #58435). For the genetic interaction analysis in the adult eye, we established fly lines carrying simultaneously hE-cad and one RNAi targeting βPS integrin (RNAi #1 obtained from BDSC #27735 and RNAi #2 obtained from BDSC #33642) or UAS-mCherry (BDSC #35787), as control for titration of the number of UAS lines in the organism. These lines were then crossed with the *GMR-Gal4* line and the appropriate progeny was selected. Eye phenotypes of at least 200 progeny flies (per condition) from three independent experiments were evaluated under a Leica compound microscope. Digital images were processed using Adobe Photoshop CS6. For further details, see Supplementary Materials and Methods.

### TCGA data analysis

RNA-seq data of 291 samples were retrieved from the supplemental data of Bass A et al. [22]. The data represent a data freeze from February 2, 2014 and is available at https://gdc.cancer.gov/about-data/publications/stad_2014. RNA-seq results include 262 gastric cancer cases and 29 adjacent non-tumour tissues (details in Supplementary Table 1 and Supplementary Materials and Methods).

### Statistical analysis

Data normality was verified with D’Agostino-Pearson omnibus test. Differences in normal distributed data were analyzed with unpaired Student’s *t* test or with one-way ANOVA, while differences in non-normal distributed data were evaluated by Kruskal–Wallis corrected with Dunn’s test for multiple comparisons. Statistical data analysis was performed using the GraphPad Prism software (version 7.04), where *P* ≤ 0.05 was considered significant.

## Results

### E-cadherin mutant cells display altered cell-matrix attachment profiles

**Fig. 1:**
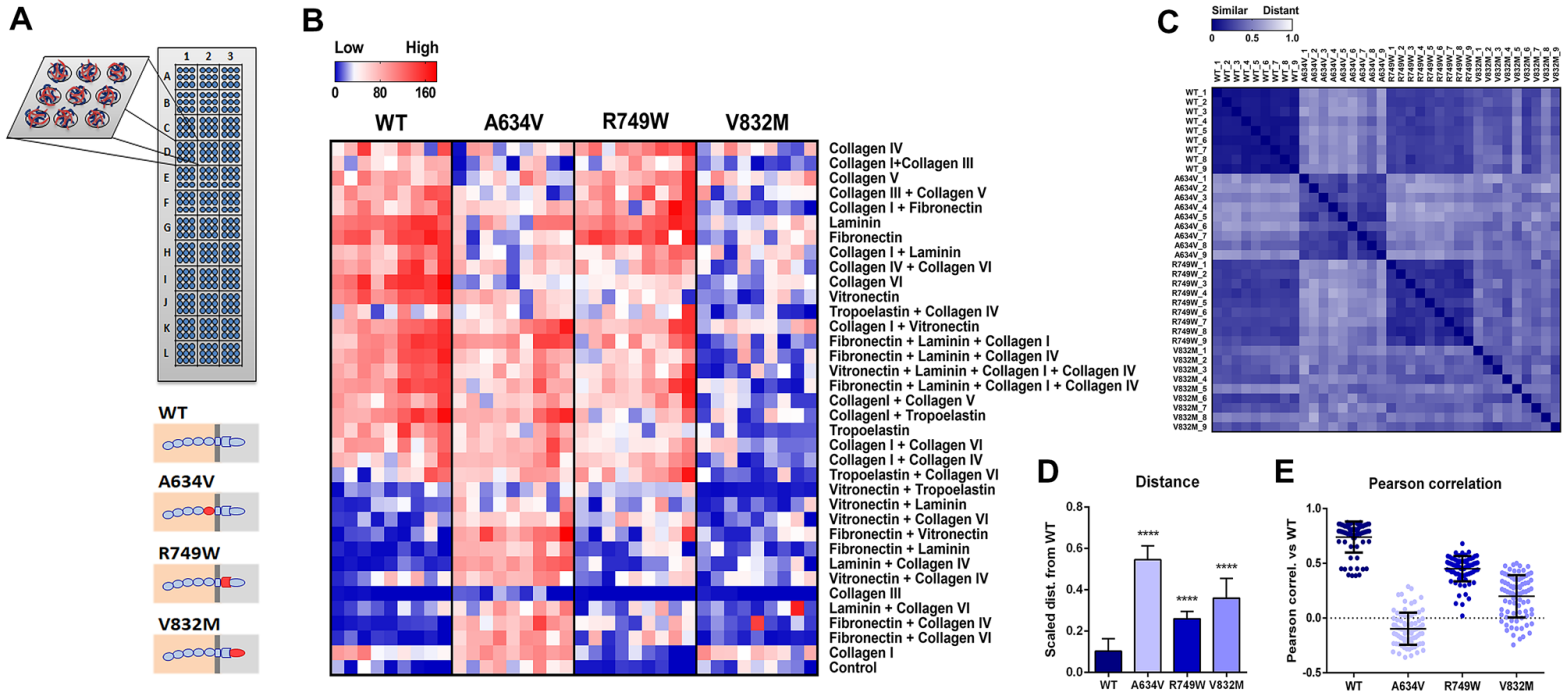
Cell-matrix interaction induced by E-cadherin variants associated with HDGC. **A** An ECM array platform was used to evaluate the adhesion profile of human gastric cells transfected with wild-type E-cadherin or with cancer-associated mutants A634V, R749W and V832M that affect, respectively, the extracellular, the juxtamembrane and the intracellular domains of the protein. **B** Heatmap showing the quantification of the adhesive abilities of AGS cells expressing the wild-type protein or the different variants. **C** Overview of matrix similarity analysis based on Pearson correlation data. Scaled distance (**D**) and Pearson correlation (**E**) between the adhesive profile of wild-type and E-cadherin mutant cells

A634V, R749W and V832M are three E-cadherin variants reported as causative in patients with HDGC [23–26]. To investigate the effects of variants associated with HDGC in cell-matrix interactions, we have used cells transfected with wild-type E-cadherin or the different variants in an array of 36 combinations of ECM proteins (Fig. 1A). The number of cells attached to each ECM spot was quantified as a direct measure of cell-matrix adhesive ability, maintaining cell maximal biological activity. In general terms, each E-cadherin mutant has a clearly distinct attachment profile (Fig. 1B). When compared with cells expressing wild-type E-cadherin, the extracellular mutant A634V shows increased adhesion for a panel of ECM combinations, which is not so striking in the juxtamembrane R749W. In contrast, the expression of the V832M intracellular variant leads to an overall trend of lower number of adherent cells.

Matrix similarity analysis, based on Pearson correlation distance, demonstrates that all variants generate a significant different pattern from that displayed by wild-type E-cadherin cells (*P* < 0.0001 for all variants, Fig. 1C–E). The A634V mutant presents the most distinct matrix from that of the wild-type reference (mean distance of 0.5458 in A634V and 0.1029 in the WT), while the R749W yields the closest adhesive activity (0.2600).

### E-cadherin mutants have common preferences regarding ECM composition

**Fig. 2:**
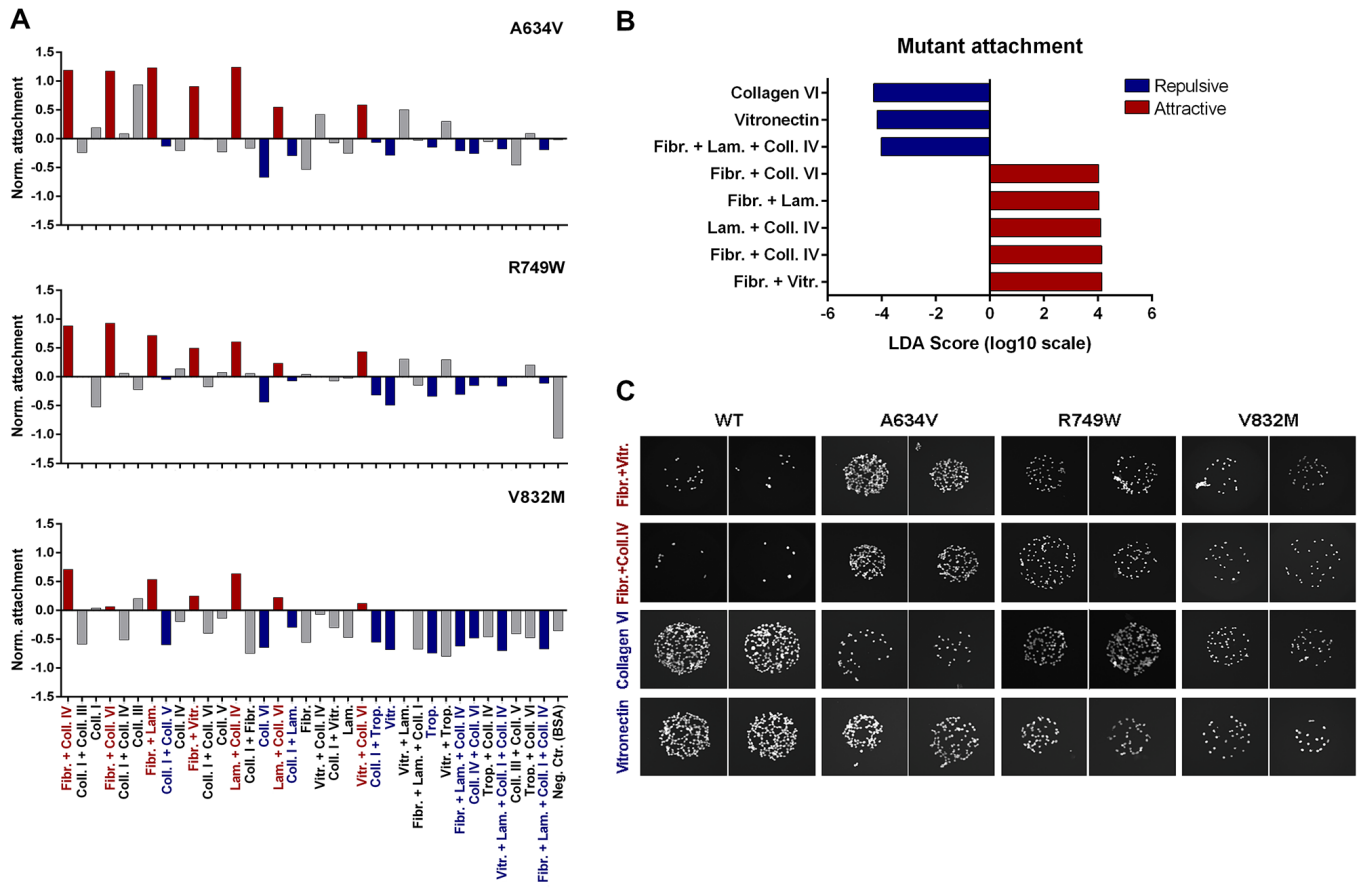
Adhesion profile of A634V, R749W and V832M E-cadherin mutants. **A** The number of attached cells was quantified and normalized for cells expressing the wild-type protein. ECM compositions consistently inducing increased adhesive abilities are depicted in red whereas repulsive substrates are presented in blue. **B** LEfSe analysis (linear discriminant analysis effect size) displaying ECM compositions that induce significant differences in matrix attachment of mutants. **C** Representative images of spots illustrating cell attachment in the most mutant-attractive and -repulsive ECM combinations

Our next aim was to determine which ECM compositions would be advantageous to E-cadherin dysfunctional cells. Although attachment ability is variable across the three E-cadherin mutants, we observed that a panel of ECM combinations induces a consistent increase in the number of adherent cells expressing any of the variants (Fig. 2A). Interestingly, there is also a set of substrates that seem to be repulsive for mutant cells, given that lower adherence is observed when compared with the wild-type.

Using linear discriminant analysis effect size (LEfSe) that couples statistical significance with biological constancy and effect size estimation, we verified that the combinations of Fibronectin + Vitronectin, Fibronectin + Collagen IV, Laminin + Collagen IV, Fibronectin + Laminin, and Fibronectin + Collagen VI promote adherence of mutant cells independently of the variant expressed (Fig. 2B). Remarkably, Collagen VI or Vitronectin on their own, as well as the mixture of Fibronectin + Laminin + Collagen IV led to a reduction of cell-matrix adhesion in all mutants but not in wild-type cells. In accordance, scarce wild-type cells adhere to spots of Fibronectin + Vitronectin or Fibronectin + Collagen IV, whereas a higher number of mutant cells adhere to these ECM enriched sites (Fig. 2C). In contrast, Collagen VI and Vitronectin are attractive substrates for wild-type expressing cells but fewer cells are able to attach in the case of E-cadherin dysfunction. Altogether, this bias on ECM preference suggests that E-cadherin dysfunction activates a specific mechano-transduction program to regulate matrix adhesion.

### E-cadherin mutant cells exert increased traction forces in attractive ECM compositions

**Fig. 3:**
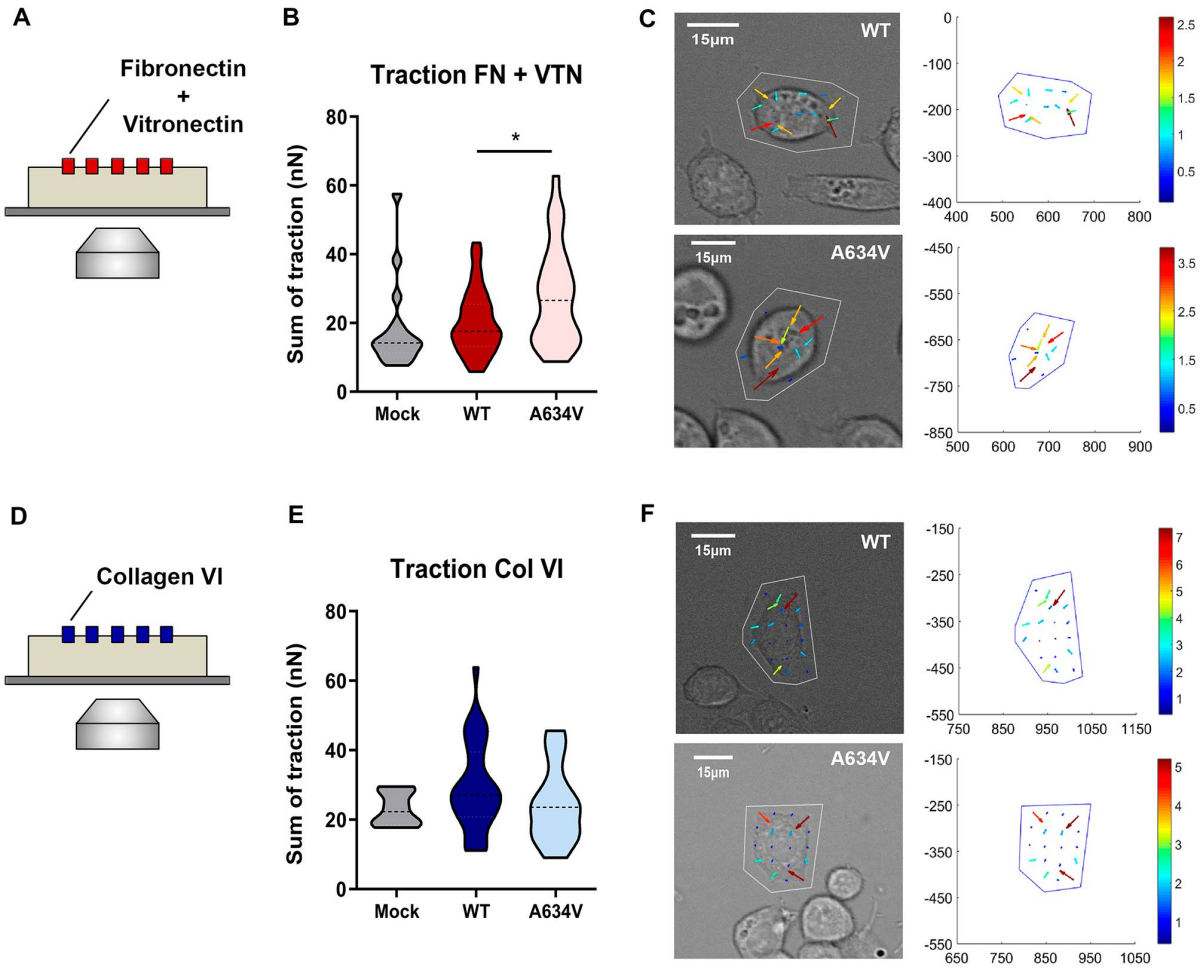
Traction force of E-cadherin mutant cells cultured on different ECM compositions. Scheme illustrating traction force microscopy on polyacrylamide gels micropatterned with Fibronectin+Vitronectin (**A**) or Collagen VI (**D**). Quantification of traction forces exerted by negative, wild-type or mutant E-cadherin cells cultured on top Fibronectin+Vitronectin (**B**) or Collagen VI (**E**). **C**, **F** Differential interference contrast (DIC) images of cells overlapped with the corresponding traction force vectors are shown on the left. Traction force vectors alone are displayed on the right panels and colored according to magnitude (nN)

It is well established that mechanical forces influence cellular and subcellular functions, including cell-matrix adhesion [27]. Therefore, we have evaluated the impact of ECM in cell mechanical loads through micropattern traction force microscopy. Traction measurements were conducted on polyacrylamide gels micropatterned with those ECM compositions inducing the most striking differences between functional and dysfunctional E-cadherin contexts. We selected the Fibronectin + Vitronectin combination to test an attractive condition, and Collagen VI to assess a repulsive situation for E-cadherin mutant cells (Fig. 3A and D).

Corroborating the previous results, cells expressing mutant E-cadherin display significant higher traction forces on gels patterned with Fibronectin + Vitronectin, when compared with cells transfected with the wild-type protein (Fig. 3B–C). In this substrate, the A634V mutant shows a mean sum of traction force of 28.0 nN, while the sum of all forces applied by cells expressing the wild-type version is 20.3 nN (*P* = 0.0171). Interestingly, AGS gastric cancer cells which are negative for E-cadherin expression present lower traction values than the wild-type model (18.0nN in Mock cells), further suggesting that cell-matrix traction forces depend on E-cadherin expression and function. Cells exhibit the opposite behaviour on top of Collagen VI, since cells expressing mutant E-cadherin present weaker traction forces than those expressing the wild-type version (24.3nN for expression of A643V and 29.4nN for expression of the wild-type, Fig. 3E–F). Of relevance, wild-type cells adhere efficiently to Collagen VI and a remarkable difference is detected in the magnitudes of the vectors generated on this ECM, when compared with those produced on top of Fibronectin + Vitronectin (WT cells: 29.4nN in Col VI vs 20.3nN in FN + VTN, respectively, *P* = 0.0007).

Overall, our results demonstrate that E-cadherin mutant cells adhere preferentially to substrates in which they can exert increased traction forces. In repulsive substrates, there is reduced cytoskeletal tension, as weaker forces are applied in micropatterned gels. This indicates that with a suitable matrix composition, mutant cells have an increased ability to engage ECM receptors to apply mechanical forces and spread into the surrounding environment.

### Integrin β1 is a candidate ECM receptor involved in the abnormal cell-matrix attachment of E-cadherin mutant cells

**Fig. 4:**
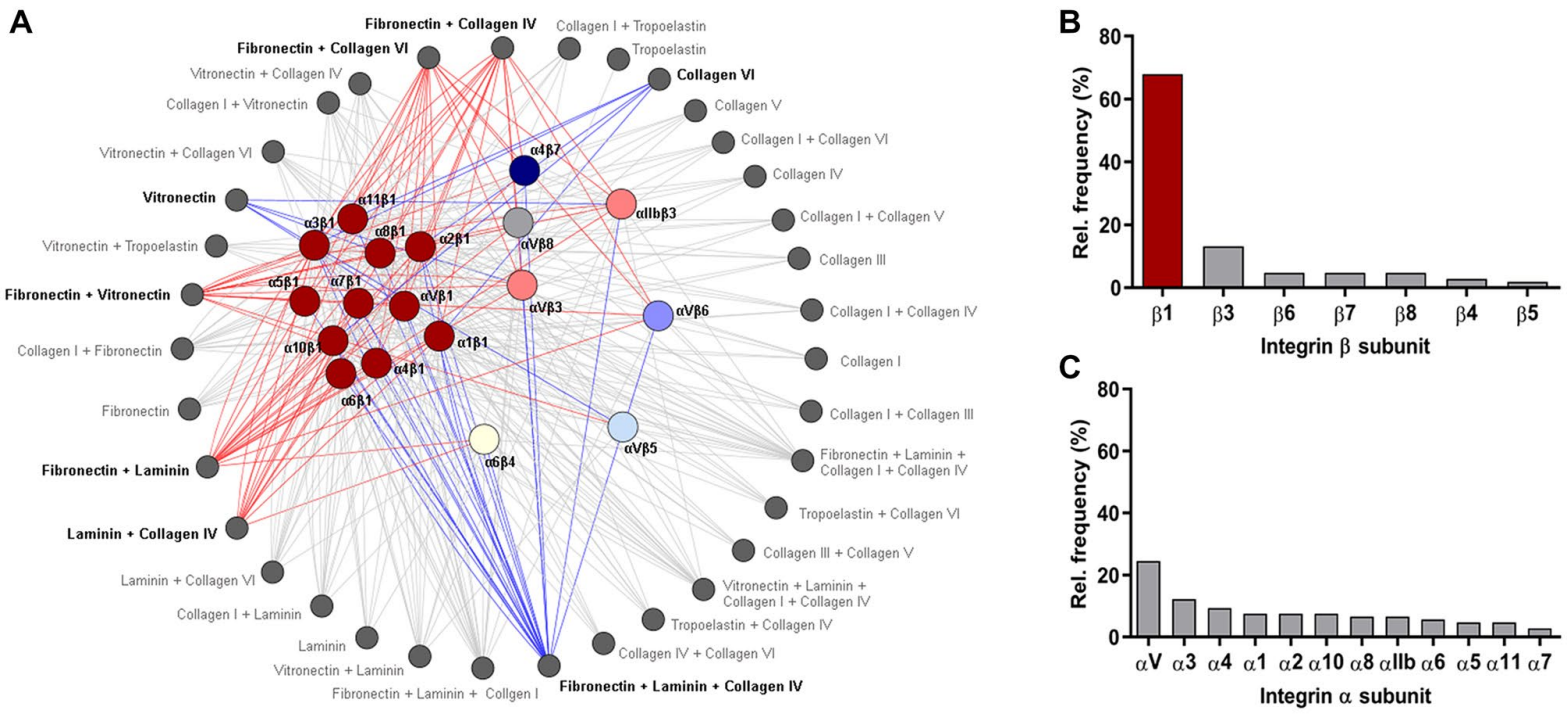
ECM receptors involved in cell-matrix attachment of E-cadherin mutant cells. **A** A network diagram illustrating possible receptors of ECM components present in the microarray was constructed using Cytoscape. ECM-interactors were collected from published data. Colored nodes indicate ECM receptors and dark grey nodes represent the 36 ECM compositions. Red edges indicate interactions of compositions that induce significant increased adhesion, and blue edges show connections of significant repulsive substrates. The interactions of ECM compositions that do not induce significant changes in cell adhesion are displayed in light grey. **B**, **C** Relative frequency of predicted interactions between integrin subunits (β and α) and the tested ECM compositions

Given that integrins are the main regulators of the cell-ECM crosstalk, we focused on the identification of those activated in E-cadherin dysfunctional cells. We implemented an ECM-integrin-guided method integrating the results of the ECM microarray profiling (Fig. 1) and a consensus view on the best-validated integrin ligands (Supplementary Table 2) [17, 18]. This method considers the ability of integrins to mediate ECM-adhesion rather than gene/protein expression features, which are usually not correlated with functional effects. Among the integrins described as interactors of the ECM compositions included in the microarray, Integrin β1 is the strongest candidate (Fig. 4A–B). Quantification of predicted molecular interactions occurring between receptors and ECM compositions inducing significant adhesion changes (identified by LEfSe analysis, Fig. 2B) pinpoints Integrin β1 as the receptor mediating 67.9% of the interactions (72/106). In contrast to the β subunit, there is no particular enrichment for the α subunit involved in ligand-binding of the 36 ECMs tested (Fig. 4C).

### Loss of Integrin β1 function rescues misexpression of human E-cadherin in vivo

**Fig. 5:**
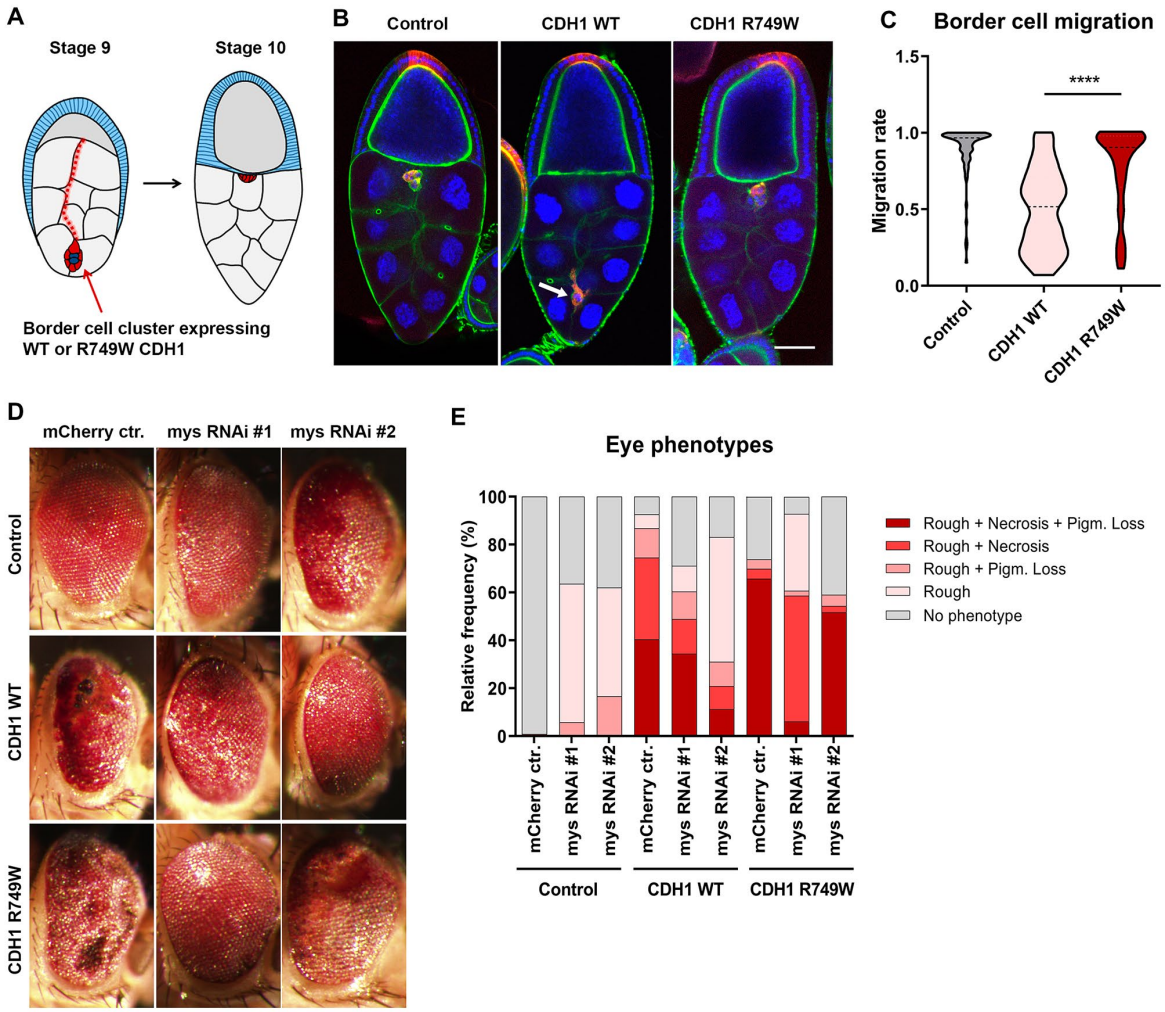
Genetic interaction between human E-cadherin and Integrin β1 in *Drosophila*. **A** Schematic representation of border cell migration in stages 9 and 10 of oogenesis. **B** Migration phenotypes in stage 10 egg chambers. Border cells are marked in red by expression of UAS driven mCherry (red), phalloidin (green) stains F-actin and the nuclei are counterstained with DAPI (blue). Scale bar = 50 µm. **C** Quantitative analysis of the border cell migration index in cells expressing *CDH1* WT (*n* = 68) and *CDH1* R749W (*n* = 71) with the *slbo-GAL4* driver, as well as in the control (*n* = 73). **D** Representative images of eye phenotypes from flies expressing the indicated UAS-driven transgenes in the developing eye with the *GMR-Gal4* driver. **E** Graph indicates the relative frequency of fly phenotypes. For each condition, *n* ≥ 200 flies analysed

To explore the importance of Integrin β1 in the context of E-cadherin dysfunction, we established an in vivo model in *Drosophila melanogaster*. We first monitored the impact of overexpressing hE-cadherin in border cell migration across the *Drosophila* germline (Fig. 5A) [28]. Expression of wild-type hE-cad disrupts border cell migration towards the oocyte (~ 49% of the expected distance, Fig. 5B–C), whereas border cells expressing the R749W mutant migrate similarly to cells expressing an inert UAS-driven transgene (UAS-mCherry).

We next misexpressed human E-cadherin in the developing *Drosophila* eye to investigate the genetic interaction with Integrin β1. For that purpose, we engineered transgenic flies expressing WT or the R749W protein concomitantly with RNAi targeting *mys*/βPS integrin, the *Drosophila* ortholog of Integrin β1 (https://flybase.org/reports/FBgn0004657.html). In contrast to flies overexpressing UAS-mCherry alone (control), the majority of flies expressing wild-type E-cadherin lose the ordered and symmetric structure of the eye, and instead show a “rough eye” phenotype frequently accompanied by necrosis and pigment loss (40%, *n* = 174/431, Fig. 5D–E). Expression of R749W further increases the penetrance of the most severe phenotype with the majority of flies presenting a rough eye with extensive necrotic areas and pigment loss (66%, *n* = 218/332). Strikingly, silencing βPS integrin by RNAi rescues the most dramatic defects for both WT and R749W E-cadherin. Specifically, only 13% of the flies co-expressing UAS-hE-cad WT and UAS-mCherry RNAi show mild phenotypes (no phenotype or rough eye), whereas these mild phenotypes are observed in 40% and 69% of flies co-expressing UAS-hE-cad WT with UAS-*mys*RNAi #1 or with UAS-*mys*RNAi #2, respectively. Likewise, the less severe phenotypes represent 26% of the cases when UAS-hE-cad R749W is co-expressed with mCherry RNAi, and increase their frequency to 39% and 41% when UAS-*mys*RNAi #1 or UAS-*mys*RNAi #2 are respectively co-expressed (Supplementary Fig. 1). Taken together, the genetic interactions detected in vivo reflect a role of Integrin β1 in cellular defects elicited by E-cadherin dysfunction.

### Integrin β1 silencing impairs invasion and increases cell–cell compaction of E-cadherin mutant cells

**Fig. 6:**
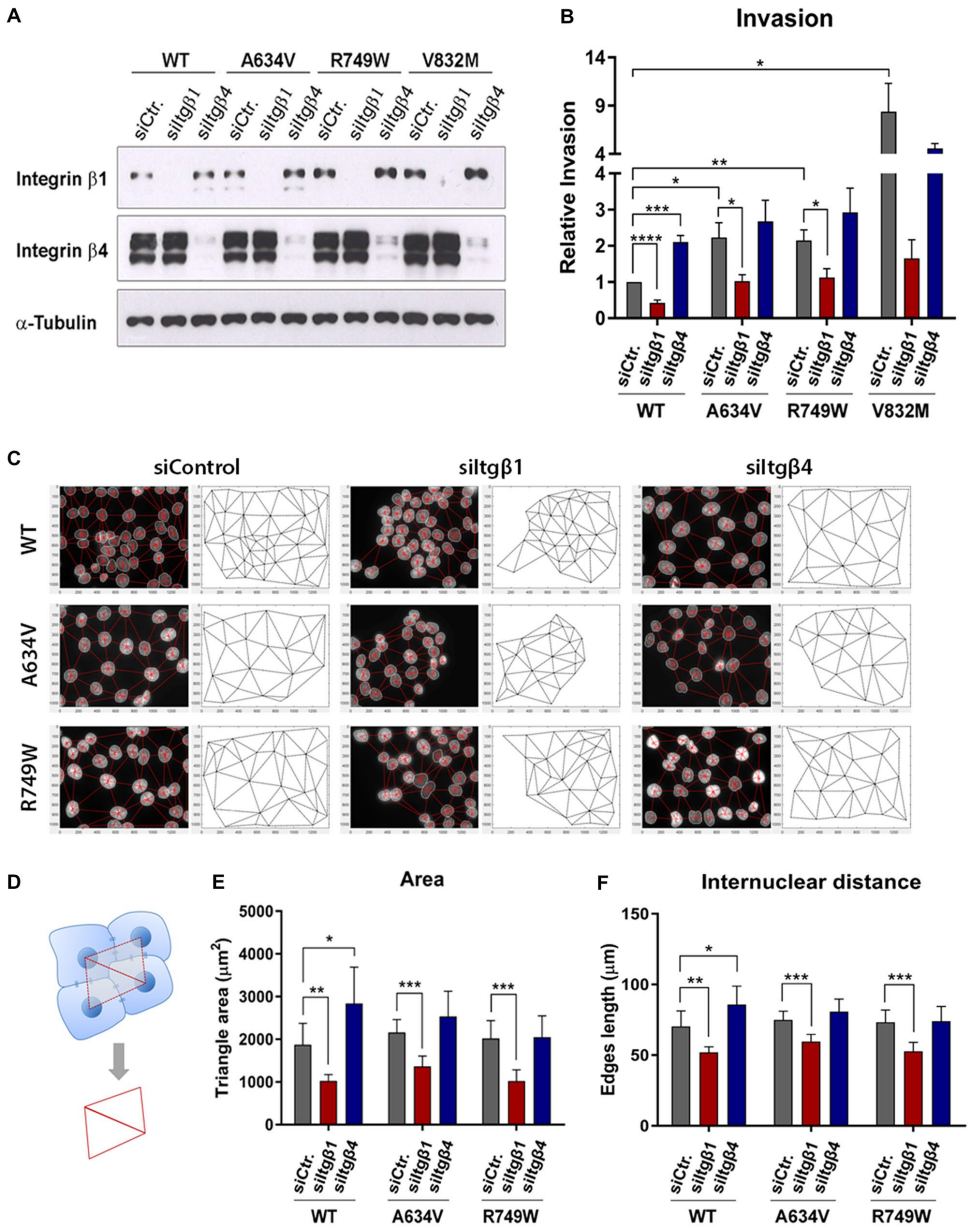
Integrin β1 functional significance in an E-cadherin mutant setting. Specific inhibition of Integrin β1 or β4 by siRNA was performed in cells stably transfected with wild-type or mutant E-cadherin. **A** Integrin β1 and Integrin β4 levels were analysed by Western Blot. α-Tubulin was used as a loading control. **B** Cell invasive ability upon integrin modulation was evaluated through matrigel invasion chambers. **C** Cellular distribution patterns elicited by integrin inhibition. Cell nuclei overlapped with the corresponding network (on the left) and final networks (on the right) are presented. **D** Scheme illustrating the measurement of cell–cell connection and cell distribution patterns through automated assembly of neighbouring nuclei. Quantitative analysis of networks regarding triangle areas (**E**) and edges length (**F**)

To study the functional relevance of Integrin β1 in human gastric cancer cells, we have performed its inhibition in AGS cells expressing either the wild-type or mutant forms of E-cadherin (Fig. 6A and Supplementary Fig. 2). Integrin β4 was also inhibited to control for specificity of cellular effects induced by knockdown of Integrin β1. Interestingly, cells expressing mutant forms of E-cadherin display significant increased levels of Integrin β1 when compared to those of the wild-type, specifically: 1.76 fold in the A634V variant (*P* = 0.0076), 2.22 fold in the R749W (*P* = 0.0011), and 1.54 fold in the V832M (*P* = 0.017). A small increase in Integrin β1 was also observed upon inhibition of Integrin β4, both in functional and dysfunctional E-cadherin cell lines (Supplementary Fig. 2).

The effect of integrin modulation on the invasive potential of cells was evaluated using matrigel invasion chambers. We observed that A634V, R749W and V832M induce an increase in the number of invasive cells, when compared with the wild-type protein (2.23, 2.15 and 8.39 fold, respectively). Nevertheless, RNAi for Integrin β1 rescues this phenotype, leading to a substantial decrease in invasion of the three E-cadherin mutants (Fig. 6B). In contrast, depletion of β4 subunit renders an increased invasive ability to cells, which is consistent with the small increase in expression of Integrin β1 detected under this experimental condition.

We then used a recent bioimaging tool based on nuclei position as a proxy for automated analysis of the spatial distribution of cells and epithelial topology [21]. Our results show that Integrin β1 downregulation generates networks formed by cells in closer proximity (Fig. 6C–F). Integrin β4 seems to play an opposite role, with cells being further away from each other and generating bigger triangles upon its inhibition. This set of experiments suggests that Integrin β1 is crucial for the initial steps of the invasive process mediated by E-cadherin dysfunction, possibly by promoting cell-matrix adhesion, cell–cell loosening and invasion of E-cadherin mutant cells.

### Loss of E-cadherin and increased Integrin β1 expression associate with tumour grade and patient overall survival

**Fig. 7:**
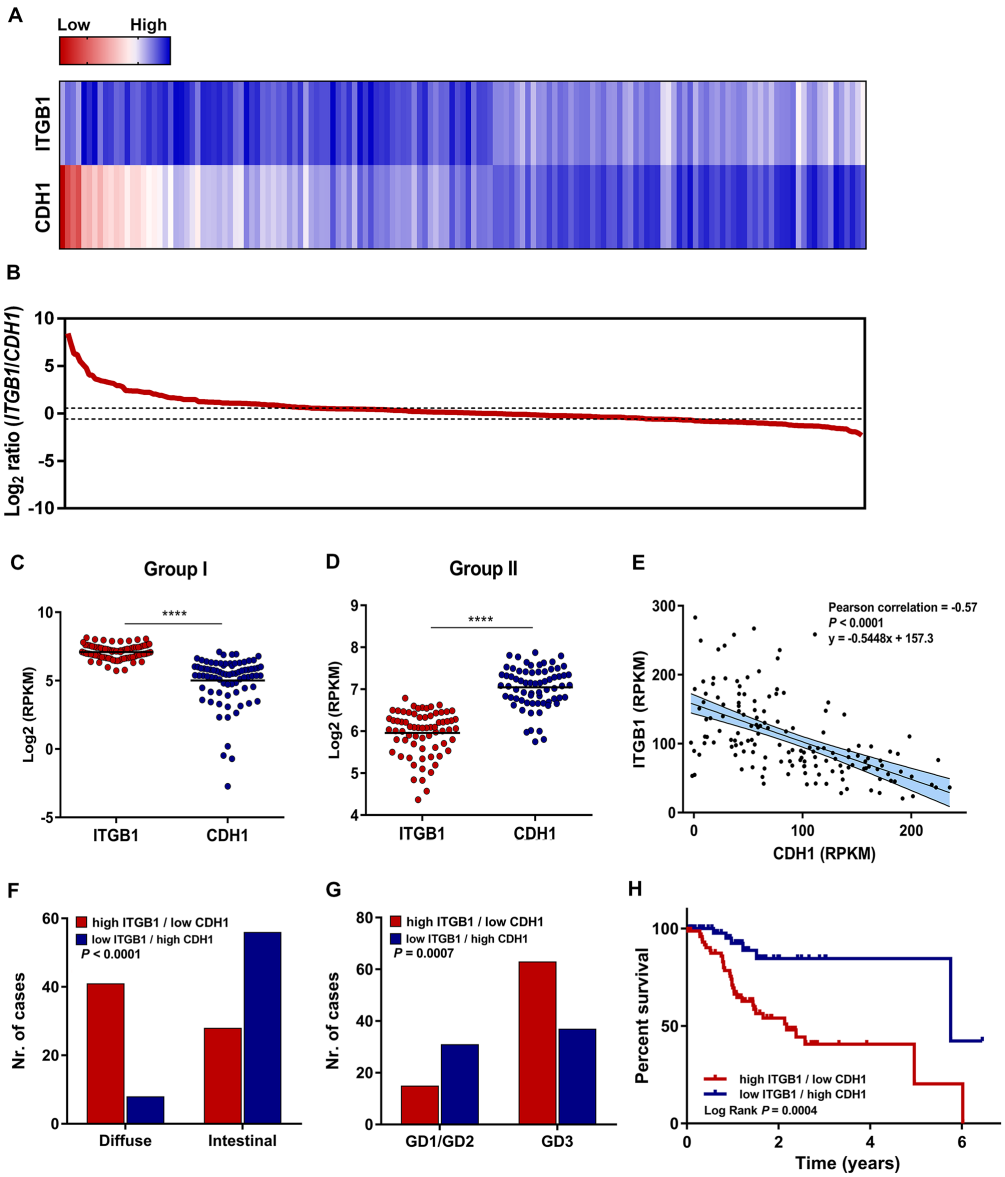
Clinical relevance of the Integrin β1/E-cadherin interplay in human gastric carcinoma samples. **A** Heatmap representing *ITGB1* and *CDH1* mRNA levels in 262 gastric cancer samples according to *ITGB1*/*CDH1* log2 ratio. **B** Graph of log2 ratio depicting two groups of samples following implementation of a 1.5-fold threshold: group I displaying high *ITGB1*/*CDH1* ratio and group II low *ITGB1*/*CDH1* ratio. **C**, **D** Differential expression of *ITGB1* and *CDH1* in the two patient groups. **E** Pearson correlation of *ITGB1* and *CDH1* reads per kilobase of transcript per million mapped reads (RPKM). Association between *ITGB1* and *CDH1* levels with tumour histological type (**F**) and grade (**G**). **H** Survival plot illustrating overall survival of patients exhibiting either high *ITGB1*/low *CDH1 or* low *ITGB1*/high *CDH1* expression

Lastly, we investigated the E-cadherin/Integrin β1 interplay using transcriptomic data of 262 gastric carcinoma samples, retrieved from TCGA [22]. By applying a threshold of 1.5-fold change (0.58 in log2) to the log2 ratio *ITGB1*/*CDH1*, we were able to discriminate two different groups of gastric carcinoma cases (Fig. 7A–B). Groups were defined as high *ITGB1*/low *CDH1* expression (group 1, *n* = 80) and low *ITGB1*/high *CDH1* expression (group 2, *n* = 69, Fig. 7C–D). Among cases included in these groups, an inverse correlation was detected between *ITGB1* and *CDH1* expression (Pearson correlation = − 0.57, *P* < 0.0001, Fig. 7E). Cases displaying high *ITGB1*/low *CDH1* expression were strongly associated with diffuse type gastric cancer and increased tumour grade (Fig. 7F–G). Moreover, these patients exhibited significantly lower overall survival, when compared with those harbouring tumours with a low *ITGB1*/high *CDH1* molecular phenotype (Fig. 7H). Additionally, using gastric cancer cell lines and available information concerning *CDH1* mutation, DNA methylation, as well as microRNA expression, we observed that the *ITGB1*/*CDH1* inverse relationship is independent of the mechanism leading to E-cadherin inactivation (Supplementary Figs. 3 and 4). These results validate the relevance of the E-cadherin/Integrin β1 pathway at the clinical level and highlight its potential as a biomarker of patient outcome.

## Discussion

In the present work, our aim was to unravel the mechanisms and key players underlying the invasive process mediated by E-cadherin mutations. Evidence from previous reports demonstrates that mechanoregulation plays an important role in epithelial tissue homeostasis, and implicates the interdependence between cadherins and integrins [13, 29, 30]. Cadherins are fundamental for intercellular junctional stability, whereas integrins are the main receptors for components of the ECM [10, 12]. Taking this into account, we postulated that E-cadherin mutations cause an imbalance at the cell-ECM interface with critical impact in cancer cell invasion.

Herein, we have addressed our aim through a novel ECM microarray approach, which is based upon cell functional activity, in contrast to gene expression or transcriptome analysis that only consider differential mRNA levels [31]. Using this platform, we have studied the (cell-ECM) adhesive function of cells expressing distinct E-cadherin variants associated with HDGC and compared it with that of the wild-type protein. We verified that different variants confer different adhesion profiles, in accordance with our previous work reporting that each E-cadherin mutation dictates a specific cell behaviour [14, 32]. Despite the marked difference between adhesion profiles, we verified that the most mutant-attractive compositions combine Fibronectin and structural ECM molecules such as Collagen IV, Collagen VI or Laminin. Fibronectin has been described to work as a bridge between structural components of the basement membrane, serving as a substrate for integrin engagement and clustering in many cell types [33–35]. Differently, Collagen IV fibers are major components within the basement membrane, and form a scaffold for association of other proteins [33, 36]. Data available concerning Vitronectin are still scarce, but it has been postulated that this adhesive glycoprotein plays a role in the provisional matrix of tumours, promoting cell adhesion and matrix degradation by binding to integrins, plasminogen activator inhibitor-1 (PAI-1) and urokinase plasminogen activator receptor (uPAR) [37]. Given the complexity of the basement membrane and the unique functions of the different ECM proteins, it is expected that cells with malignant potential do not adhere to matrices of a single component such as Collagen VI or Vitronectin. We may speculate that, under these conditions, mutant cells are able to survive in an anchorage independent manner.

It is well known that the biophysical properties of the ECM regulate tensional homeostasis [9]. We have therefore measured traction forces exerted by wild-type and mutant cells on attractive and repulsive ECM substrates. Higher traction forces were detected in matrices to which cells adhere more efficiently, and while mutant cells exert increased tension on Fibronectin + Vitronectin, a lower mechanical input is observed in Collagen VI. The opposite effect was seen in wild-type cells. Of note, Fibronectin and Vitronectin are ligands for RGD-binding integrins (integrins with affinity for Arg-Gly-Asp sequence), whereas the receptors for collagen belong to a distinct integrin class, named αA-domain-containing integrins [17]. This suggests that an integrin switch may occur from a competent to an incompetent E-cadherin context, involving activation of the most promiscuous class of integrins.

By combining ECM adhesion profiles and available data on specific ECM receptors, we identified Integrin β1 as the receptor most likely involved in the response of E-cadherin dysfunctional cells. Integrin β1 overexpression was already described in gastric cancer and associated with vascular invasion and lymph node metastasis [38, 39]. The β1 subunit was found to be crucial for peritoneal dissemination of human gastric carcinoma, both in patient samples and xenograft models [40–42]. Nevertheless, a causal relationship between E-cadherin loss of function and Integrin β1 activity remained elusive.

A possible E-cadherin/Integrin β1 linkage was addressed in vivo by the establishment of transgenic fly lines expressing wild-type and mutant human E-cadherin, in which we modulated the expression of the *Drosophila* ortholog of Integrin β1 (βPS). Our experiments demonstrated that integrin expression contributes to the severe effects of human E-cadherin misexpression, since depletion of βPS integrin reduces the prevalence of the stronger phenotypic defects in eye morphology. We then elucidated the biological significance of Integrin β1 in E-cadherin defective cells by taking advantage of our in vitro cancer cell model. We found that cells expressing E-cadherin missense variants indeed exhibit increased levels of Integrin β1. Remarkably, Integrin β1 downregulation increased cell–cell compaction and induced a marked decrease in the invasive ability of mutant cells. These results corroborate data showing that Integrin β1 expression induces scattering of mouse epithelial cells through regulation of the cadherin-catenin complex, as well as remodelling of the actin cytoskeleton [43]. In addition, it is known that Integrin β1 inhibition blocks E-cadherin internalization, leading to increased intercellular adhesion [30].

Finally, we found an inverse correlation between E-cadherin and Integrin β1 expression in a series of 262 gastric carcinoma cases, confirming that E-cadherin loss triggers deregulation of cell-matrix interactions regardless of the mechanism leading to *CDH1* inactivation. Cases presenting low E-cadherin expression levels exhibited high levels of integrin and were associated with increased tumour grade and poor patient overall survival. In agreement with the present findings, our group has previously reported overexpression of Laminin γ2 and decreased E-cadherin in gastric cancer cell lines, *Drosophila* models and primary tumours [44]. Laminin γ2—a major basement membrane component—allows E-cadherin defective cells to survive and invade, contributing to gastric carcinogenesis [44]. Importantly, β1 integrins (α3, α6 and α7) are receptors for laminins.

A critical issue for future research should cover the characterization of the ECM proteome and its regulators, or “matrisome”, in normal, pre-malignant and malignant gastric epithelia, which remain largely unexplored and would complement our understanding of the oncogenic cell-ECM affair.

## Conclusion

This work shed light on the molecular machinery implicated in the invasive process mediated by E-cadherin mutations. We provided evidence that E-cadherin dysfunctional cells activate specific integrins, namely Integrin β1, for efficient adhesion and dissemination through the ECM. We propose that Integrin β1 can be used in the clinics as a new predictive biomarker of gastric cancer progression.

## Supplementary Information

Below is the link to the electronic supplementary material.

Supplementary file1 (DOCX 33 KB)

Supplementary file2 (DOCX 686 KB)

Supplementary file3 (DOCX 27 KB)
