# Supplementary material for: Integrin β1 orchestrates the abnormal cell-matrix attachment and invasive behaviour of E-cadherin dysfunctional cells
Source: Gastric Cancer. 2021 Sep 5;25(1):124–37. doi: 10.1007/s10120-021-01239-9 (PMC8732838; doi:10.1007/s10120-021-01239-9)
Supplement: Supplementary file 1 — Supplementary file1 (DOCX 33 KB) [file 10120_2021_1239_MOESM1_ESM.docx]

**Supplementary MaterialS and Methods**

*Cell culture and transfection* – AGS cell line (gastric adenocarcinoma, ATCC number CRL-1739) was cultured in RPMI medium (Gibco, Invitrogen) supplemented with 10% fetal bovine serum (HyClone, Perbio) and 1% penicillin/streptomycin (Gibco, Invitrogen). Cells were incubated at 37°C under 5% CO_2_ humidified air. Transfections were performed using Lipofectamine 2000 (Invitrogen), according to the manufacturer's recommendations. For overexpression experiments, 1µg of DNA of vectors encoding the wild-type protein or the A634V, R749W and V832M variants, as well as the empty vector was used. Transfected cells were selected by antibiotic resistance to blasticidin (5μg/ml; Gibco, Invitrogen). In the case of specific inhibition with siRNAs, a SMARTpool of 4 different siRNAs targeting Integrin β1 or Integrin β4 mRNAs was purchased from Dharmacon and prepared according to the manufacturer’s instructions. Nonsilencing siRNA duplexes (Dharmacon) were used as a negative control. Depletion efficiency was maximum at 48h using 200nM siRNA. At the end of each transfection, putative cytotoxic effects were evaluated, analysing cell viability.

*Micropattern Traction Microscopy* – Micropattern traction microscopy was performed as previously described [[1](#_ENREF_1), [2](#_ENREF_2)]. Briefly, a solution of 125 μg/ml AlexaFluor-488 (Thermo Fisher) tagged Fibronectin and 125 μg/ml Vitronectin or a solution of 250 μg/ml AlexaFluor-488 tagged Collagen VI was patterned onto a plasma-treated glass coverslip. A grid of 2 μm diameter dots with 6 μm separation from centre to centre was used for patterning. The coverslip patterning was then transferred onto a 6.7 kPa stiffness polyacrylamide gel. The polyacrylamide gel precursor solution contained 0.002% acrylic acid N-hydroxysuccinimide ester (NHS) (Sigma), which allows covalent bonding of the protein pattern onto the gel surface during polymerization. The micropatterned gel was seeded with 5x10^4^ cells, 24h before experimental assays. Imaging was performed using an Olympus IX881 microscope with a Hamamatsu Orca R2 camera. Images were taken every 5 min for 1h. All experiments took place in an environmental chamber at 37ºC, 70% humidity and 5% CO_2_. Custom MATLAB (MathWorks) scripts were used for analysis of cell images and fluorescent grid patterns. The program determines the displacement vector (**u**) of the geometrical centre of the dots and calculates the corresponding traction force vectors (**F**) through the formula **F** = π*Ea***u**/(2 + ν ‒ ν^2^). Therein, *a* = 1 μm is the radius of the dot markers, *E* is the Elastic modulus of the polyacrylamide gel (6.7 kPa) and ν = 0.445 is the Poisson’s ratio of the gel substrate [[1](#_ENREF_1)]. The magnitude of F is calculated for each force acting on the cell at 5 min time intervals. The sum of all traction force magnitudes was obtained and then time-averaged over 1 h. This feature was used as a metric of the traction field magnitude [[2](#_ENREF_2)].

*Western blotting* – Lysates were obtained by scraping cells in cold Catenin Buffer [1% Triton X-100 (Sigma) and 1% IGEPAL CA-630 (Sigma) in PBS], enriched with a cocktail of phosphatase (Sigma) and protease inhibitors (Roche). Protein concentration was assessed using a modified Bradford assay (Bio-Rad). For analysis, 15μg of total protein were eluted in sample buffer, separated in 10% SDS-polyacrylamide gels (SDS–PAGE), and electroblotted onto Hybond ECL membranes (Amersham Biosciences). Membranes were blocked in 5% non-fat milk and 0.5% Tween-20 in PBS for 1h, and thereafter immunoblotted with antibodies against E-cadherin (1:2500, Clone HECD1, Invitrogen), Integrin β1 (1:1000, BD Biosciences), Integrin β4 (1:1000, Cell Signaling) and α-Tubulin (1:10000, Sigma). The secondary antibodies sheep anti-mouse or donkey anti-rabbit HRP-conjugated (Amersham Biosciences) were then incubated, followed by detection with ECL reagents (Bio-Rad). Protein bands were quantified by densitometry using the Quantity One Software (Bio-Rad).

*Matrigel invasion assays* – Cell invasive abilities were assessed using matrigel invasion inserts suitable for 24-well-plates (Corning BioCoat). Matrigel chambers were hydrated with α-MEM medium for 1h. 500µl of a cellular suspension of 5 x 10^4^ cells/ml (containing 2.5 x 10^4^ cells) were then plated in each chamber. The plate was incubated at 37ºC in a humidified atmosphere with 5% CO_2_. Upon 24h of incubation, non-invasive cells were removed from the upper part of the filter with a pre-wet ‘cotton swab’. Filters were washed in PBS and fixed in ice-cold methanol for 15 minutes. Vectashield medium with DAPI (Vector Laboratories) was applied for filter mounting. Total number of invasive nuclei present in the bottom of each filter was counted under a Leica DM2000 microscope.

*Drosophila strains and genetic manipulations* – The plasmid CDH1pENTR 221 (Clone ID: IOH46767, Invitrogen) and its R749W mutant form were subcloned onto the pPW-attB destination vector using LR clonase II-mediated recombination. pPW-attB was generated by removal of the MYC encoding region present in pPMW-attB (Addgene plasmid #61814). The different transgenes were then inserted into the attP40 landing site via PhiC31 site-specific transgenesis (BestGene Inc), placing wild-type and mutant E-cadherin under the same genetic environment, thus ensuring similar transgene regulation.

*Ovary preparation and F-actin staining* – *Drosophila* ovaries were dissected in Schneider’s Insect Medium (Sigma) supplemented with 10% FBS. Fixation was performed in 4% paraformaldehyde in PBS for 20 minutes. Alexa Fluor 488 phalloidin (ThermoFisher) was used for F-actin staining. Ovaries were washed with 0.05% Tween-20 in PBS and mounted in Vectashield with DAPI (Vector Laboratories). Fixed tissue was imaged using an inverted laser scanning confocal microscope (Leica TCS SP5 II, Leica Microsystems) and processed using Leica Application Suite (LAS) software. To measure the migration index in stage 10 egg chambers, images were processed using Fiji [[3](#_ENREF_3)], and the position of the border cell cluster was normalized to the total distance from the anterior part of the egg chamber to the oocyte.

*TCGA data analysis –* RNA-seq data of 291 samples was retrieved from the supplemental data of Bass A *et al*. [[4](#_ENREF_4)]. The data represents a data freeze from February 2, 2014 and is available at https://gdc.cancer.gov/about-data/publications/stad_2014. RNA-seq results include 262 gastric cancer cases and 29 adjacent non-tumour tissues (details in Supplementary Table 2). Reads per kilobase of transcript per million mapped reads (RPKM) of *CDH1* and *ITGB1* were extracted from cancer samples. RPKM values were log2 transformed and the log2 ratio between the two genes was calculated. Two groups of cases were identified using log2 ratio higher than 0.58 or lower than -0.58, representative of a 1.5-fold difference: one group expressing high *ITGB1* and low *CDH1* levels; and the other exhibiting low *ITGB1* and high *CDH1* expression. Both groups were compared across several clinicopathological parameters, namely age, gender, neoplasm histological grade, Lauren classification, WHO classification and overall survival. Differences in gene expression were assessed using unpaired T-test. Correlation between *ITGB1* and *CDH1* expression was evaluated using Pearson’s correlation coefficient, whereas associations with categorical variables were determined using Fisher’s exact test. Differences in group overall survival were analysed using survival curves followed by log-rank (Mantel-Cox) test. Available information on *CDH1* mutation, DNA methylation, as well as microRNA expression in gastric carcinoma samples were also collected and examined.

**References**

1. Polio SR, Rothenberg KE, Stamenovic D, Smith ML. A micropatterning and image processing approach to simplify measurement of cellular traction forces. Acta biomaterialia **2012**; 8:82-8.

2. Xu H, Donegan S, Dreher JM, et al. Focal adhesion displacement magnitude is a unifying feature of tensional homeostasis. Acta biomaterialia **2020**; 113:372-379.

3. Schindelin J, Arganda-Carreras I, Frise E, et al. Fiji: an open-source platform for biological-image analysis. Nat Methods **2012**; 9:676-82.

4. Cancer Genome Atlas Research N. Comprehensive molecular characterization of gastric adenocarcinoma. Nature **2014**; 513:202-9.
