# Supplementary material for: Integrin β1 orchestrates the abnormal cell-matrix attachment and invasive behaviour of E-cadherin dysfunctional cells
Source: Gastric Cancer. 2021 Sep 5;25(1):124–37. doi: 10.1007/s10120-021-01239-9 (PMC8732838; doi:10.1007/s10120-021-01239-9)
Supplement: Supplementary file 2 — Supplementary file2 (DOCX 686 KB) [file 10120_2021_1239_MOESM2_ESM.docx]

**
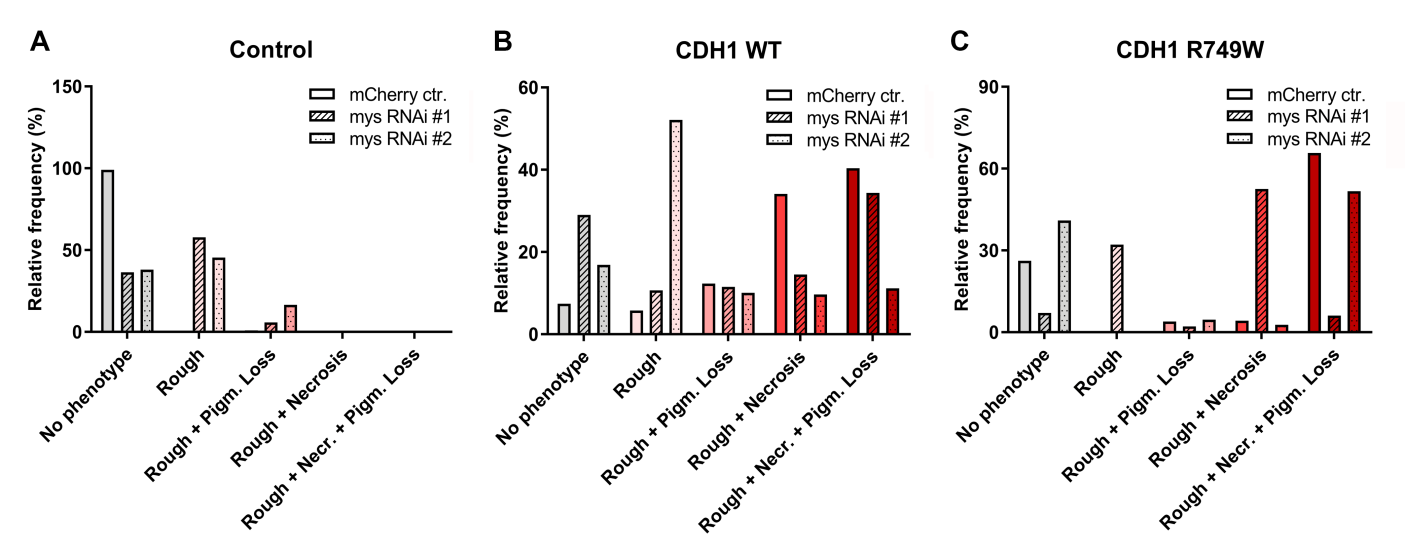
**

**Supplementary Figure 1. Phenotypic effects of βPS integrin silencing in wild-type and mutant E-cadherin contexts.** Relative frequency of eye phenotypes in control (A), UAS-hE-cad WT (B) or UAS-hE-cad R749W (C) flies co-expressing either RNAi targeting βPS integrin or UAS-mCherry.


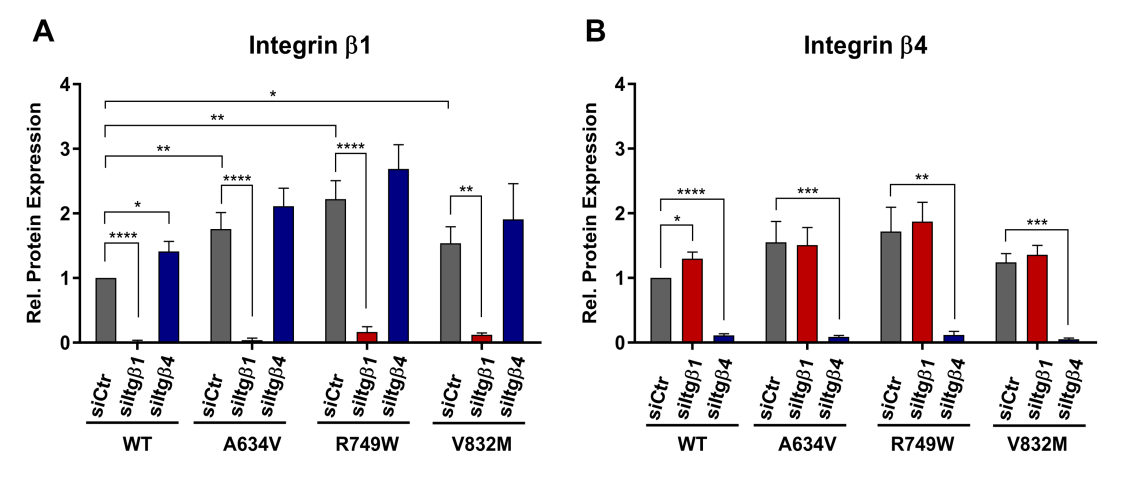


**Supplementary Figure 2. Efficiency of Integrin β1 and Integrin β4 knockdown in wild-type and mutant E-cadherin cell lines.** Protein levels were analysed by Western Blot. Band intensity was quantified and normalized to wild-type cells treated with non-targeting siRNA. Graphs represent intensity average + SE of Integrin β1 (A) and Integrin β4 (B).

**
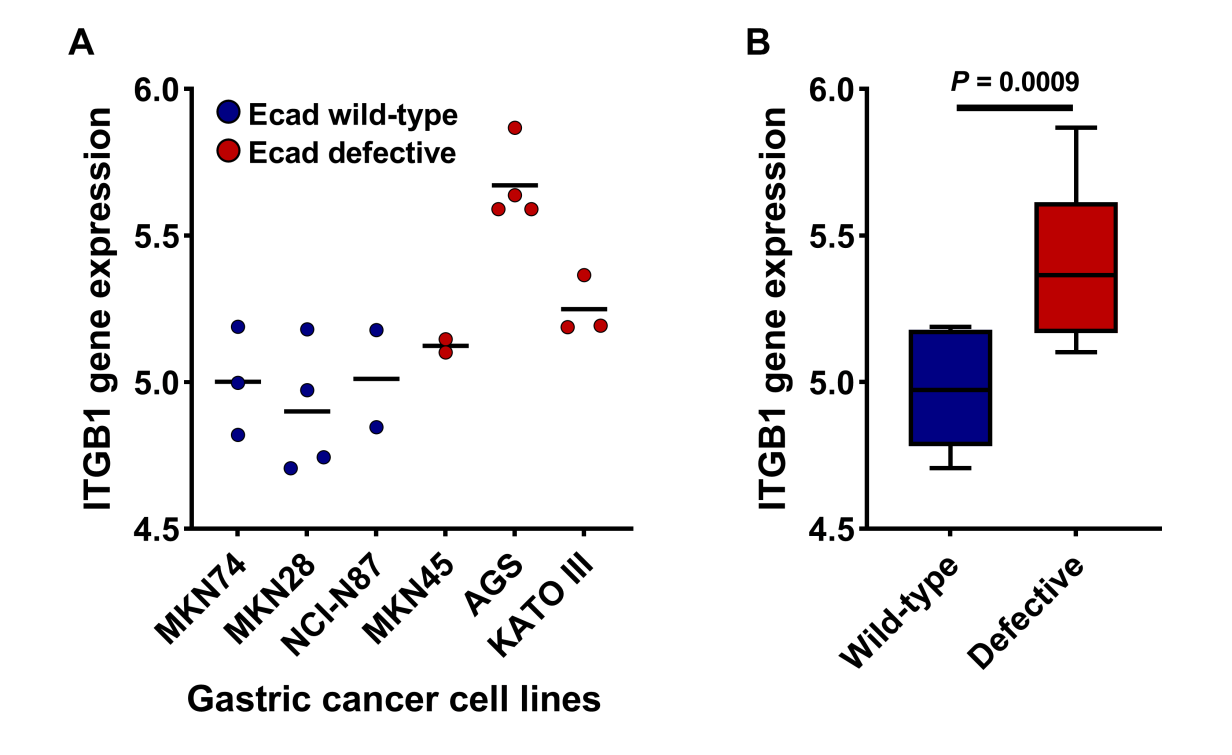
**

**Supplementary Figure 3. *ITGB1* expression in gastric cancer cell lines with distinct *CDH1* status.** (A) *ITGB1* mRNA levels were retrieved from the NCBI Gene Expression Omnibus repository (Accession number GSE146361). Gene expression was evaluated through HumanHT-12 v3.0 Expression BeadChip array (Illumina). Gastric cancer cell lines displaying wild-type *CDH1* include MKN74, MKN28 and NCI-N87 [[1](#_ENREF_1)]. In contrast, MKN45, AGS and KATO III comprise the set of E-cadherin defective cells. MKN45 harbours an 18b.p. deletion in exon-intron 6 boundary, whereas Kato III carries a G to A base substitution of the last 3´nucleotide of exon 7. AGS cells are characterized by complete E-cadherin silencing due to the presence of a truncating mutation along with loss of heterozygosity (LOH). (B) Graph displaying a comparison of *ITGB1* expression between wild-type E-cadherin cell lines and those presenting E-cadherin dysfunction.


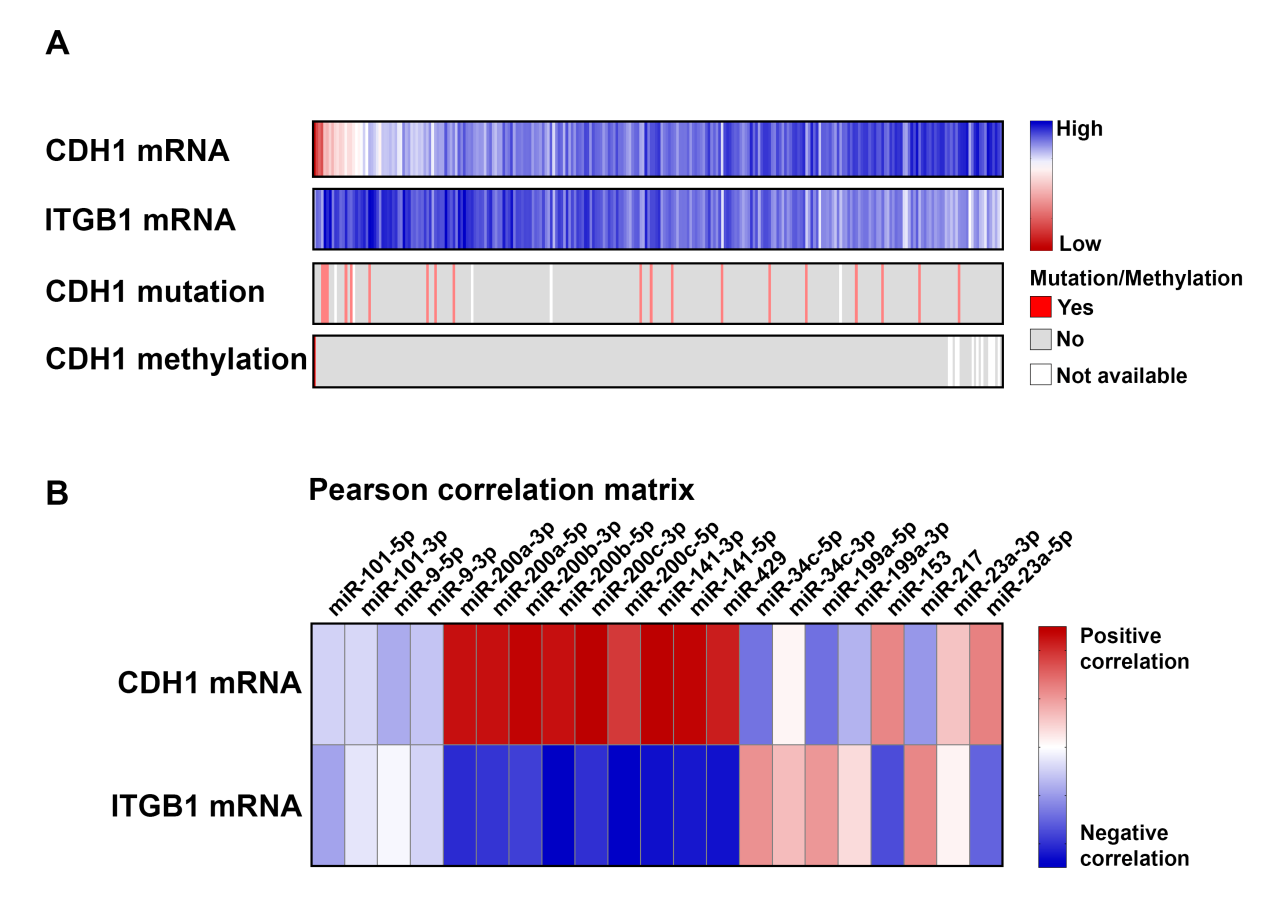


**Supplementary Figure 4: E-cadherin/Integrin β1 crosstalk is independent on the mechanism of *CDH1* inactivation.** (A) Heatmap representing *CDH1* and *ITGB1* mRNA levels in 262 gastric cancer samples according to *ITGB1*/*CDH1* log2 ratio. The corresponding *CDH1* mutation and methylation status are indicated. (B) Pearson correlation of microRNAs, reported as *CDH1* regulators [[2](#_ENREF_2)], with *CDH1* or *ITGB1* expression.

**References**

1. Carneiro P, Moreira AM, Figueiredo J, et al. S100P is a molecular determinant of E-cadherin function in gastric cancer. Cell communication and signaling : CCS **2019**; 17:155.

2. Rossi T, Tedaldi G, Petracci E, et al. E-cadherin Downregulation and microRNAs in Sporadic Intestinal-Type Gastric Cancer. International journal of molecular sciences **2019**; 20.
