# Supplementary material for: Integrin β1 orchestrates the abnormal cell-matrix attachment and invasive behaviour of E-cadherin dysfunctional cells
Source: Gastric Cancer. 2021 Sep 5;25(1):124–37. doi: 10.1007/s10120-021-01239-9 (PMC8732838; doi:10.1007/s10120-021-01239-9)
Supplement: Supplementary file 3 — Supplementary file3 (DOCX 27 KB) [file 10120_2021_1239_MOESM3_ESM.docx]

**Supplementary Table 1.** **Clinicopathological features of 291 gastric cancer samples retrieved from Cancer Genome Atlas Research Network [**[**1**](#_ENREF_1)**].** Available parameters were analysed according to *ITGB1* and *CDH1* gene expression.

|  |  |  |  | **Characterization according to *ITGB1* and *CDH1* expression** | | | | | | |
| --- | --- | --- | --- | --- | --- | --- | --- | --- | --- | --- |
|  | **Total** | |  | **+ *ITGB1* / - *CDH1*** | |  | **- *ITGB1* / + *CDH1*** | |  | ***P*-value** |
| **Type of sample** | **n** | **%** |  | **n** | **%** |  | **n** | **%** |  |  |
| Primary Tumor | 262 | 90.0% |  | 80 | 92.0% |  | 69 | 80.2% |  | 0.029* |
| Solid Tissue Normal | 29 | 10.0% |  | 7 | 8.0% |  | 17 | 19.8% |  |  |
|  |  |  |  |  |  |  |  |  |  |  |
| **Gender** |  |  |  |  |  |  |  |  |  |  |
| Male | 163 | 62.2% |  | 48 | 60.0% |  | 48 | 69.6% |  | 0.235* |
| Female | 99 | 37.8% |  | 32 | 40.0% |  | 21 | 30.4% |  |  |
|  |  |  |  |  |  |  |  |  |  |  |
| **Neoplasm histologic grade** | |  |  |  |  |  |  |  |  |  |
| Gx | 5 | 1.9% |  | 2 | 2.5% |  | 1 | 1.4% |  | 0.008# |
| G1 | 6 | 2.3% |  | 2 | 2.5% |  | 4 | 5.8% |  |  |
| G2 | 82 | 31.3% |  | 13 | 16.3% |  | 27 | 39.1% |  |  |
| G3 | 169 | 64.5% |  | 63 | 78.8% |  | 37 | 53.6% |  |  |
|  |  |  |  |  |  |  |  |  |  |  |
| **Laurén classification** |  |  |  |  |  |  |  |  |  |  |
| Diffuse | 65 | 24.8% |  | 41 | 51.3% |  | 8 | 11.6% |  | < 0.001# |
| Intestinal | 170 | 64.9% |  | 28 | 35.0% |  | 56 | 81.2% |  |  |
| Mixed | 16 | 6.1% |  | 6 | 7.5% |  | 4 | 5.8% |  |  |
| Not specified | 11 | 4.2% |  | 5 | 6.3% |  | 1 | 1.4% |  |  |
|  |  |  |  |  |  |  |  |  |  |  |
| **WHO classification** |  |  |  |  |  |  |  |  |  |  |
| Tubular | 120 | 45.8% |  | 18 | 22.5% |  | 40 | 58.0% |  | < 0.001# |
| Mucinous | 16 | 6.1% |  | 5 | 6.3 |  | 1 | 1.4% |  |  |
| Papillary | 21 | 8.0% |  | 1 | 1.3% |  | 13 | 18.8% |  |  |
| Poorly cohesive | 65 | 24.8% |  | 41 | 51.3% |  | 8 | 11.6% |  |  |
| Mixed | 16 | 6.1% |  | 6 | 7.5% |  | 4 | 5.8% |  |  |
| Not specified | 24 | 9.2% |  | 9 | 11.3% |  | 3 | 4.3% |  |  |
|  |  |  |  |  |  |  |  |  |  |  |
| **Survival** |  |  |  |  |  |  |  |  |  |  |
| Alive | 180 | 68.7% |  | 45 | 56.3% |  | 50 | 72.5% |  | 0.039# |
| Deceased | 82 | 31.5% |  | 35 | 43.7% |  | 18 | 26.1% |  |  |
|  |  |  |  |  |  |  |  |  |  |  |
|  | **Mean** | **SD** |  | **Mean** | **SD** |  | **Mean** | **SD** |  |  |
| **Age**** | 66 | 11 |  | 64 | 11.6 |  | 67 | 10 |  | 0.097§ |
|  |  |  |  |  |  |  |  |  |  |  |
| *Fisher’s exact test; #Chi-square test; §T-test; **Three cases did not have available data concerning age. | | | | | | | | | | |

**Supplementary Table 2. ECM proteins and corresponding integrin receptors.** Putative integrin receptors for ECM proteins used in the array were identified based upon a consensual network of validated interactions [[2](#_ENREF_2), [3](#_ENREF_3)].

| **ECM protein** | **Integrin receptors** |
| --- | --- |
| Collagen I | α10β1, α2β1, α1β1, α11β1, α3β1 |
| Collagen III | α10β1, α2β1, α1β1, α11β1, α3β1 |
| Collagen IV | α10β1, α2β1, α1β1, α11β1, α3β1 |
| Collagen V | α10β1, α2β1, α1β1, α11β1, α3β1 |
| Collagen VI | α10β1, α2β1, α1β1, α11β1, α3β1 |
| Fibronectin | αIIbβ3, αVβ3, αVβ6, αVβ1, α5β1, α8β1, α4β1, α4β7, α3β1, αVβ8 |
| Laminin | α3β1, α6β1, α6β4, α7β1, α1β1, α2β1, α10β1 |
| Vitronectin | αIIbβ3, α8β1, αVβ3, αVβ5, αVβ1 |
| Tropoelastin | αVβ3 |

**References**

1. Cancer Genome Atlas Research N. Comprehensive molecular characterization of gastric adenocarcinoma. Nature **2014**; 513:202-9.

2. Humphries JD, Byron A, Humphries MJ. Integrin ligands at a glance. J Cell Sci **2006**; 119:3901-3.

3. Jin H, Varner J. Integrins: roles in cancer development and as treatment targets. Br J Cancer **2004**; 90:561-5.
